# Statistical Analysis and Optimisation of Data for the Design and Evaluation of the Shear Spinning Process

**DOI:** 10.3390/ma14206099

**Published:** 2021-10-15

**Authors:** Sandra Puchlerska, Krzysztof Żaba, Jarosław Pyzik, Tomasz Pieja, Tomasz Trzepieciński

**Affiliations:** 1 Department of Metal Working and Physical Metallurgy of Non-Ferrous Metals, Faculty of Non-Ferrous Metals, AGH University of Science and Technology, Al. Mickiewicza 30, 30-059 Kraków, Poland; krzyzaba@agh.edu.pl; 2 Sabre Polska Sp. z o.o., Wadowicka 6D, 30-415 Kraków, Poland; jaro.pyzik@gmail.com; 3 Pratt&Whitney Rzeszów S.A., Hetmańska 120, 35-001 Rzeszów, Poland; tomasz.pieja@pwrze.utc.com; 4 Department of Manufacturing and Production Engineering, Faculty of Mechanical Engineering and Aeronautics, Rzeszów University of Technology, al. Powstańców Warszawy 8, 35-959 Rzeszów, Poland; tomtrz@prz.edu.pl

**Keywords:** superalloy, design of experiments, 3D scanning, statistical optimisation

## Abstract

This work proposes a research method that is a scheme that can be universally applied in problems based on the selection of optimal parameters for metal forming processes. For this purpose, statistical data optimisation methods were used. The research was based on the analysis of the shear spinning tests performed in industrial conditions. The process of shear spinning was conducted on the components made of Inconel 625 nickel superalloy. It was necessary to select the appropriate experimental plan, which, by minimising the number of trials, allowed one to draw conclusions on the influence of process parameters on the final quality of the product and was the starting point for their optimisation. The orthogonal design $2_{\mathrm{III}}^{3-1}$ is the only design for three factors at two levels, providing non-trivial and statistically significant information on the main effects and interactions for the four samples. The samples were analysed for shape and dimensions using an Atos Core 200 3D scanner. Three-dimensional scanning data allowed the influence of the technological parameters of the process on quality indicators, and thus on the subsequent optimisation of the process, to be determined. The methods used proved to be effective in the design, evaluation and verification of the process.

## 1. Introduction

Shear spinning is a method of rotating forming of components on a rotating forming block using a roller. This technique is recommended for small and medium series production of axisymmetric products [1,2]. Plastic deformation of the material in the process of shear spinning occurs by direct action of the pressing rollers on a small contact area with the material. The method is used for the production of products made of carbon and stainless steels, non-ferrous metal alloys and “difficult-to-form” materials [3,4,5]. The shear formed products have increased strength and hardness, with favourable microstructure directionality. The advantages of shear spinning include: high dimensional accuracy, the possibility of application to large deformations, low tooling costs, and high surface quality [6,7].

There are many methods of analysing and optimising the shear spinning process. The first analytical models of the shear spinning process were developed by Dröge in 1954 [8] to calculate the tangential forces during the formation of cylindrical parts. The author points out that this is a time-consuming method. Another model was developed by Avitzur and Yang [9] for shear forming. They calculated the tangential force assuming a pure shear state for the sheet under the roll. In the model, they focused the feed, roller radius and wall angle. Another model of shear spinning proposed by Kalpakcioglu [10], assumes that the rectilinear deformation with plane deformation allows to estimate the tangential force. The model was verified by Kobayashi [11], who proposed an additional assumption of the bending mechanism. Moreover, the radial and axial force components in the contact area of the material with the roller were estimated. Sortais et al. published a slightly different proposal [12]. They extended the pure shear model to aspects related to flange deformation. Hayama and Murota [13] proposed two models of spinning, assuming axial deformation. In both models, three force components were calculated on the basis of the pressure distribution on the roller, and the unknown friction coefficient was determined by fitting the analytical force calculations to the experimental results. Forces at low feed rates are predicted with high accuracy. In contrast to the axisymmetric models, Wang et al. [14] proposed an asymmetric analytical model for predicting the axial force. Kim et al. [15] obtained a relatively accurate model using experimentally determined material flow curves. Chen et al. [16] calculated all three components of force. The main feature that distinguishes this model from the previous ones is the inclusion of over-spinning as a parameter. Joorabchian and Slater [17] proposed models that adopt axisymmetric and plane strain to estimate the upper limit of the tangential force during shear spinning. Estimates coincide with experimental results. Two analytical models for predicting deformation during conventional spinning are described in the paper of Quigley and Monaghan [18]. In both studies, considerations were made based on changes in the geometry and the constancy of volume. Comparing the authors’ suggestions with experiments provides useful insight into the deformation mechanisms in both spinning and shear forming.

The first numerical model of shear spinning was developed by Alberti et al. [19]. Importantly, the ability of the numerical model to predict geometry, strains and loads in the process has been demonstrated, although the authors do not verify their values. A similar approach to the axisymmetric elasto-plastic model was developed by Liu et al. [20]. They investigated the effect of different tool paths on deformation and stresses in the process. A more refined model was proposed by Mori and Nonaka [21], who artificially introduced shear stresses into the oscillometric model. The main advantage of the Mori and Nonaka model compared to the previous proposals is the ability to predict the material gain before the roll and also to use this information to predict failure in the form of cracks. Three-dimensional numerical models are designed to improve accuracy but require longer computational times. The first three-dimensional model was proposed by Quigley and Monaghan [22], who used an intermediate solution algorithm with an elasto-plastic material model. In order to shorten the computation time, the authors attempted both adaptive mesh densification and parallel processing. The authors emphasize the importance of accurate modelling of the distance between the roller and the forming block. They indicate that a slight change in distance has an intense effect on the stresses arising under the tool. Moreover, the calculated forces were shown to be the same as those calculated by Wang et al. [14], with the difference that Wang et al. calculated the forces for the three-step process, while Quigley and Monaghan modelled only part of the first roll pass. Another model of hot shear spinning was developed by Klocke and Wehrmeister [23] to optimise the location of the laser beam and the heated zone. Their analysis was carried out separately, respectively in the Abaqus and Deform simulation programs. The intermediate elastoplastic model for hot process was used by Lu et al. [24] to study over-spinning and under-spinning flange bending. Two elasto-plastic shear spinning models were developed by Kleiner et al. [25] and Klimmek et al. [26]. They used them to study stresses and folds in conventional spinning. Zhan et al. [27] developed a direct elasto-plastic model of the cone spinning process to investigate stress, strain and thickness distributions and predict the effect of feed on tool forces and geometric tolerances of manufactured parts. The authors report that the maximum deviation of the geometry predicted by the model largely coincides with the experiments, with the maximum error of 22%. Gao et al. [28] built a model that investigates the influence of the parameters of the hot spinning process on microstructure, stress state and on damage evolution. The authors concluded that the inner surface of workpiece with the largest voids volume fraction is the place with the greatest potential of fracture. Moreover, the authors suggested an optimal configuration of processing parameters for hot forming of tubes.

To predict process failure caused by cracks or folding in the free surface, the authors propose several experimental approaches. The first is the approach published by Kegg [29], who tried to predict process failure caused by fracture in the rotary crush process. On the basis of experimental observations, he proposed an empirical relation in order to establish the shear spinning of a given material. The Kegg approach was extended by Hayama and Tago [30] to include the occurrence of a failure mode of the process in the form of folds. Additionally, the wall-cracking capacity of the intermediate was discussed by Kegg in [29]. Hayama et al. [31] used strain gauges installed on both sides of the blank, close to the edge of the sheet, to study the deformation and folding of the flange in the process of shear spinning. In publication [32], by capturing the deflection of weld line in flow forming of welded tube, the characteristic and mechanism of circumferential twist in flow forming was investigated. The weld line turned out to be useful for the evaluation of the geometrical characteristics of the circumferential deformation.

One of the methods of assessing the quality of the shear spinning process and products is statistical optimisation, using experimental data. Kawai [33] used a computer database to design shear spinning process. The database consists of approximately one hundred industrial cases that can be searched by dimension, shape and material for both input materials and products. The database was extended by Kawai et al. [2] to also include knowledge of faults and final product quality in the process. A similar approach was presented by Kleiner et al. [25], who applied the DoE (design of experiments) method to determine the influence of process parameters on the initiation of folding in the conventional spinning process. To analyse the forming forces, surface roughness and product geometry, three different approaches based on the DoE methodology were identified. The first of them was proposed by Chen et al. [3], using the DoE in conjunction with regression analysis to establish the empirical relationship between the process parameters and the forming forces and surface roughness in the case of the shear spinning. The second approach, proposed by Kunert et al. [34] uses DoE in conjunction with case-based reasoning to optimise geometry and geometric tolerance in conventional spinning.

In general, the aim of the experimental investigations of shear spinning presented in this paper is to obtain information on the relationship between the input factors (process parameters) and the output quantities (describing, for example, the characteristics of the final product). Such relations are usually presented in the form of a regression function approximating the results of tests on the basis of input factors. A properly conducted experiment, after a correct analysis of the results, allows the significance of the influence of input factors and their possible interactions to be determined giving the researcher the opportunity to select the most important factors and to eliminate the insignificant ones. This information can be used to describe the mechanism of a given phenomenon or to optimise the process parameters. Before planning the experiments, the type and number of dependent variables (responses) and of input variables, their ranges and disturbance factors must be determined. This stage is an important step in the planning of the experiment because the omission of important factors, an excess of them, or the presence of unresolved interrelationships may result in incorrect conclusions from the analysis. Having such test characteristics, an appropriate set of samples is selected. The choice of an experiment design depends on the purpose and specification of the research. The intuitive approach is to select a complete experiment design in which the trials correspond to all possible parameter configurations. Such an approach, however, entails an exponential increase in the number of trials (for a design with n factors on k levels, the number of trials is k^n^). Often, an experiment design prepared in this way is impossible to implement due to the duration of the experiments, high costs or the number of available samples. Therefore, statistical planning of experiments is commonly used. Such an approach allows the number of required trials to be significantly reduced while obtaining sufficient information to build an accurate approximation of the dependent variables. The most frequently used designs are factorial, orthogonal and central composite [35]. An orthogonal design minimises the number of trials that are necessary, among which there is any combination of the values of any two factors. A method which uses orthogonal designs is the widely used Taguchi method of process optimisation [36], applied to research on the spinning process by Wang [37], and Sangkharata and Dechjarerena [38]. Analysis of the results obtained following this experimental design enables one to identify the main effects, their significance and interactions between the factors. The main effect is the influence of a specific factor on the dependent variable, independent of other factors. In contrast, in the case of factor interactions, the effect of one factor on the dependent variable varies depending on the value of the other factor. In the analysis of the shear spinning process, Kunert et al. [34] obtained interactions between the working radius of the roll and the distance between the roll and the forming block, as well as interactions between the working radius of the roll and the resulting tool path.

**Figure 1:**
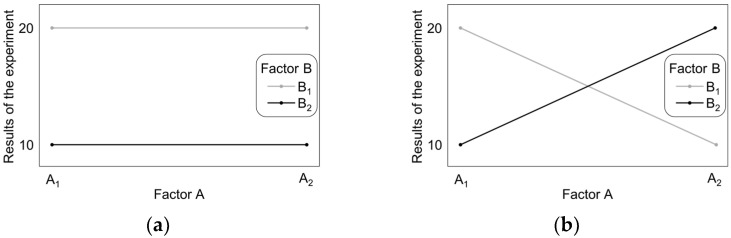
Interaction graph for experiment T_1_: (**a**) no interaction between factors A and B and (**b**) interaction of factors A and B.

For a complete two-factor experimental design with two levels (labelled as a 2^2^ design), a simple way to identify the main effects and interactions is to plot the main effects. Figure 1 shows the results of theoretical experiments T_1_ and T_2_, for factors A and B changing at two levels A_1_ and A_2_, and B_1_ and B_2_, respectively [36].

When comparing the marginal means for a specific factor, it is seen that there exists a significant difference between the means related to the individual values of factor B and the fact that there are no differences between the means related to factor A. This indicates that the main effect is the result of factor B. For experiment T_2_, there are no differences between the marginal means, however the level of factor A changes the influence of factor B on the results. This means that factors A and B interact. In the graph of effects, the main effect is visible as a parallel line for the results of experiments at individual levels (Figure 1a), while the crossing lines indicate the interaction of factors (Figure 1b).

In the case of orthogonal plans, and in general, fractional factorial designs, attention should be paid to the so-called resolution of the design, describing the occurrence of the interactions involved. Table 1 shows the experimental design for three variable factors at two levels $2_{\mathrm{III}}^{3-1}$ with resolution III. This case involves the effects ${\;X}_{1\;}$ i and interaction ${\;X}_{2}X_{3}$, effect ${\;X}_{2\;}$ and interaction ${\;X}_{1}X_{3}$ as well as the effect $X_{3\;}$ i and interaction ${\;X}_{1}X_{2}$. This means that it is impossible to distinguish the main effect from the effect of the interaction of the remaining factors. Thus, the design shown in Table 2 does not give a clear answer concerning the main effects and interactions. Further analysis or improvement of the resolution of the plan is required, for example by extending it. However, this is not a problem to analyse when the interactions involved are insignificant. Designs with resolution III are often used to study the overall system response and to pre-select significant factors [39].

The problem of optimal selection of parameters is a frequent issue when planning production processes and developing technologies. The selection of process parameters that allow a final product that meets the given requirements to be obtained is one of the principal aims of the technologist. Shear spinning parameters are selected based on previous experience, analytical models of the mechanics of the forming process, as well as the results of numerical simulations. For the shear spinning process, many authors, both in theoretical and experimental works [18,40], described the influence of individual process parameters on the occurrence of various types of defects and the quality of the product. Conversely, a problem in the practical application of these results is the need to simultaneously optimise all the quality indicators. The main obstacle is the divergent optima of indicators, where the optimal responses (for example, the desired values of the product features) are obtained for different values of a specific parameter, or there are cases when a change of a parameter increases the value of a certain indicator. The aim of the multidimensional optimisation of parameters is to find values such that the final product quality meets all the requirements within the set tolerance. The approach used in the study, using the concept of the preference function, was originally proposed by Harrington [41] and modified by Derringer and Suich [42]. The last-mentioned optimisation variant was successfully used in the work of Kunert et al. [34] to obtain the optimal set of parameters for the shear spinning process.

This work proposes a research method which is a scheme that can be universally applied in problems involving the selection of parameters for the shear spinning process. For this purpose, statistical data optimisation methods were used. The optimisation of process parameters was based on an analysis of the results of the industrial shear spinning process of drawpieces made of Ni-based Inconel 625 superalloy. It was necessary to select the appropriate type of the experimental plan, which by minimising the number of trials allowed the effect of the process parameters on the final product quality to be determined and was the starting point for their optimisation. The use of 3D scanning and non-contact and non-destructive technology permitted the influence of the technological parameters of the process on quality indicators, and thus on the subsequent optimisation of the shear spinning process, to be determined.

## 2. Materials and Methods

**Figure 2:**
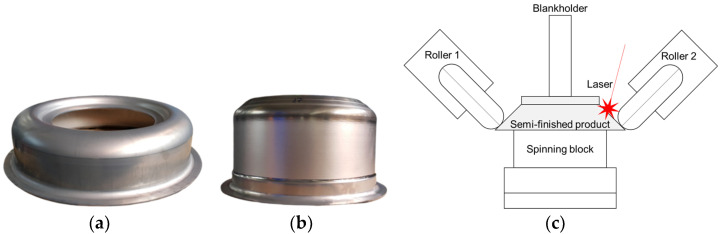
Preformed drawpiece (**a**), finished product (**b**) and stand (**c**).

The material used for the tests consisted of drawpieces (Figure 2a) made of chromium–nickel–molybdenum Inconel 625 superalloy (ATI Flat Rolled Products, Pittsburgh, PA, USA). The test material was subjected to a uniaxial tensile test in room temperature on the Zwick/Roell Z050 machine (Zwick/Roell, Ulm, Germany) in three directions, in relation to the sheet rolling direction: 0°, 45°, and 90°. The stress–strain curve was taken, and the Lankford coefficient was calculated. Microstructure analysis was also performed using a scanning electron microscope (SEM) Hitachi SU-70 (Hitachi Ltd., Tokyo, Japan). The grain size has been calculated. The drawpieces, with an external diameter of 400 mm, were formed from 1 mm thick sheet metal. The drawpieces were manufactured on a hydraulic press by Schuler Pressen GmbH SMG-260 (Schuler Group, Göppingen, Germany) with a maximum pressing force of 100 T. The preformed drawpieces (Figure 2a) were subjected to the shear spinning process with laser heating in industrial conditions on a Leifeld stand equipped with a semiconductor laser from Laserline GmbH (Laserline GmbH, Mülheim-Kärlich, Germany). The drawpieces were mounted on a spinning block and pressed against a blankholder (Figure 2c). Two symmetrically spaced rollers with a working radius R8 were used for forming. Forming parameters: rotational speed and feed were adjusted. The heating temperature was 600 °C. The laser heating parameters were as follows: the angle of incidence of the laser beam was 135°, the shape of the laser spot was rectangular with dimensions of 10 mm × 40 mm, the laser power was 5 kW. No lubricant was used during forming, only coolant was applied to the spinning block. An element of the combustion chamber of a jet engine was the end product of the forming process (Figure 2b).

In order to obtain an axisymmetric product with a varying thickness of the drawpiece wall, experiments were designed to verify the optimal shear spinning parameters. The boundary conditions and key process parameters were determined from the results of extensive cold and laser heated shear spinning studies. It was decided to conduct investigations for three factors at two established levels. The factors selected were the rotational speed of the spinning block (R), the feed rate of the roller (S) and the heating of the material (H). 

A $2_{\mathrm{III}}^{3-1}$ experiment was planned. As a starting value for determining the parameter levels, the parameters of the reference test P_0_ were adopted as: R_0_ = 220 rpm and S_0_ = 20 mm/min, H_0_ = 0. The experimental design was based on levels increased in relation to the reference values by 15% for the rotational speed, by 20% for the feed rate and by 50% for both rotational speed and feed rate. The differentiated increase for the values of the first level is a result of the need to examine the influence of the feed coefficient (f), defined as the ratio of feed rate to rotational speed. For equal percentage increments of the reference levels, the value of the feed coefficient would be the same in all cases. There are two levels of heating factor: the use of heating (1) or no heating (0). The levels of factors are shown in Table 3. The trial plan and the reference test conditions are shown in Table 4.

The $2_{\mathrm{III}}^{3-1}\;$ experiment, as designed, provides information about the main effects, the possible interactions and their influence on individual continuous quality indicators. Information on the main effects and interactions is obtained from the plot of effects for marginal means versus factor value. Continuous quality indicators were used for the geometrical evaluation of the products. In the Derringer and Suich method [43], for the dependent variables Y_i_, which are quality indicators, it is possible to introduce their dependence from k factors (parameters) X_j_ and an error $\epsilon_{i}$:

(1) $$\epsilon_{i}:Y_{i}=f_{i}\left(X_{1}\ldots X_{k}\right)+\epsilon_{i}$$

In practice, the accurate value of coefficient $f_{i}$ is often unknown; thus, the estimator $\hat{Y_{i}}$ is introduced which is obtained by the regression method. Models of proportional chances for qualitative variables and generalised linear models for quantitative variables are used in this work considering the interaction between factors c_ij_:

(2) $$\hat{Y_{i}}=c_{0}+c_{i}\overset{N}{\underset{i=1}{\sum }}X_{i}+c_{\mathrm{ij}}\overset{N}{\underset{i=1}{\sum }}\overset{N}{\underset{j=1}{\sum }}X_{i}X_{j}$$

Each estimator $\hat{Y_{i}}$ has a preference value $d_{i}\in \left[0,1\right]$. The d_i_ preference determines how much the value of $\hat{Y_{i}}$ is preferred, making it a value from 1 (most preferred) to 0 (not accepted). Based on the value of d_i_, the so-called global preference function G for the variables $\hat{{\;Y}_{i}}$. G function is defined as the weighted geometric mean of the preference value d_i_ (Equation (3)):

(3) $$G={\left(\overset{N}{\underset{i\;=1}{\prod }}d_{i}^{w_{i}}\right)}^{\frac{1}{k}}$$

where $k=\overset{N}{\underset{i\;=1}{\sum }}w_{i\;}{\mathrm{and}\;w}_{i}>0$.

The G function defined in this way is an indicator of overall quality. If any of the factors d_i_ is zero, G also becomes zero. In turn, G-values close to 1 indicate that values of d_i_ are also close to 1 (most preferred). It means that the value of Y_i_ is close to optimal. Subsequent maximisation of the G-function allows the process parameters that result in the best overall product quality to be found, while the individual indicators are maintained within the tolerance range.

**Figure 3:**
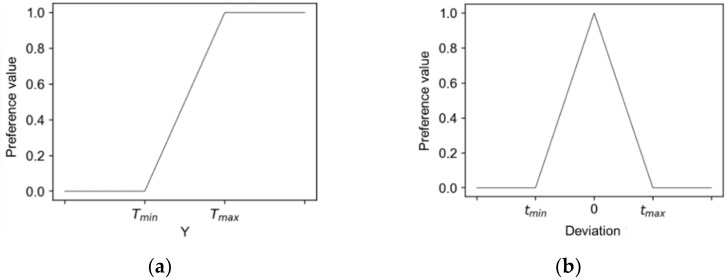
One-sided preference function (**a**), two-sided preference function (**b**).

When the value of preference d_i_ increases (or decreases) with change invariable Y_i_, the goal of optimisation is to maximise (or minimise) the response Y_i_ using a one-way preference transformation. This transform function is monotonous, reaching the value 0 below a certain fixed value T_min_ and value 1 above a certain fixed value T_max_. In the interpretation, this means that it is most desirable to achieve at least the T_max_ value, while values below T_min_ are not accepted and the preference for them is 0. The one-sided transform function can be expressed by the following function (Figure 3a):

(4) $$G={\left(\overset{N}{\underset{i\;=1}{\prod }}d_{i}^{w_{i}}\right)}^{\frac{1}{k}}$$

A two-sided transform function is used to obtain a response value close to a certain value in the interval, called the target value. The preference function then reaches 1 for that value and is set to zero outside the specified tolerance range. The rate of growth of the preference rating is controlled by the r and l coefficients. The coefficient r is responsible for the rate of increase of the transform function, and thus the rate of increase in preferences along with the approach to the desired value of S_i_. For a certain response Y, its target value (the most preferred) T and the tolerance range $\left[T-t_{\min},\;T+t_{\max}\right]$, the transformation of the Y value to the preference value d is determined by the following function (Figure 3b):

(5) $$T\left(x\right)=\{\begin{array}{cc} 0, & x\;<\;T-t_{\min}\;lub\;x\;>\;T+t_{\max}\\ \;{\left(\frac{x-\left(T-t_{\min}\right)}{t_{\min}}\right)}^{l}, & x\;\in \;\left[T-t_{\min},T\right)\\ \;{\left(\frac{\left(T+t_{\max}\right)-\;x}{t_{\max}}\right)}^{r}, & x\;\in \;\left[T,T+t_{\max}\right] \end{array}$$

For both a one-sided and two-sided transformation, one can use a spliced transform function. The spliced function is often used to transform qualitative variables.

Thanks to the transformation of preferences and the global preference function, the task of optimising the parameters in terms of the responses of quality indicators comes down to the problem of optimising the scalar function of arguments that are the preferences of the indicators of the specific parameters. The task of optimisation for the global preference function G, indicators Y_i_, their transformation T_i_, and the set of parameters P is therefore as follows (Equation (6)):

(6) $$\max\;G\left(T_{1}(Y_{1}\;\left(p\right)),\ldots ,\;T_{n}\left(Y_{n}\left(p\right)\right)\right)\;\mathrm{dla}\;p\in P$$

Among the many methods of solving such a problem, a frequently used method is grid search (GS), which involves searching the parameter space, estimating the value of the objective function, its optimisation cost and validation with the use of a cross test. In the case of a large number of parameters, or a wide range of their values, it is often impossible to exhaustively search for combinations of parameters. The most often used to optimise parameters are, inter alia, gradient methods, heuristic methods, or a randomised procedure for selecting parameter values [43]. In this paper, due to the small number of parameters, an exhaustive search and the cost function determined by the general preference function G (Equation (3)) are used.

After the technological tests, the shape and dimensions of the components were tested using an Atos Core 200 (GOM) optical 3D scanner (GOM, Braunschweig, Germany). STL files were generated from 3D scans. In the next step, the STL files were compared with the product CAD file in order to verify the deviations of the geometry from the nominal shape using GOM Inspect software (GOM, Braunschweig, Germany). Overall comparisons of the 3D surface scans were made with the CAD model and the following surface quality indicators (SURF_G) were generated: maximum positive surface deviation (MAX), maximum negative surface deviation (MIN), average surface deviation (AVG) and mean square deviation of the surface (SIGMA).

**Figure 4:**
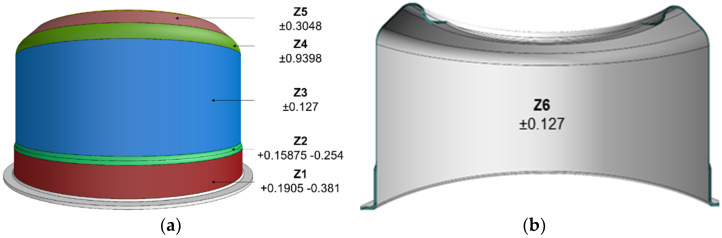
Characteristic zones of the component with specific thicknesses and variations in toler-ance, (**a**) **Z1**–**Z5**, (**b**) **Z6**.

Overall comparisons of the sheet thickness on 3D scans were also made with the CAD model and quality indicators (TH_G) were generated: maximum positive thickness deviation, maximum negative thickness deviation, average thickness deviation, mean square thickness deviation and percentage indicator of out-of-tolerance measurements (TOTALFAIL). In the next step, five component zones (**Z1**–**Z5**) with different dimensional tolerances for specific nominal thicknesses of component (Figure 4) were designated.

The tolerances were determined on the basis of the technical drawing of the component. As in the case of overall comparisons, for each Z zone, comparisons of the thickness from the 3D scans were made with the CAD model and the following quality indicators were generated (TH_Zn, where n is the number of the component zone): maximum positive thickness deviation, maximum negative thickness deviation, average thickness deviation, mean square thickness deviation, and percentage indicator of out-of-tolerance measurements. Then, two component zones **Z1** and **Z6** (inner component surface) were designated which had different thickness and diameter tolerances. Then, comparisons of the 3D scan surfaces were also made with the CAD model and quality indicators were generated (SURF_Zn, where n is the number of the component zone): maximum positive surface deviation, maximum negative surface deviation, mean surface deviation, mean square deviation of the surface. All data were interpreted in the 2σ range. For overall indicators, TH_G and SURF_G, a positive (negative) tolerance was determined as the maximum (minimum) values from specific zone tolerances. For the TOTALFAIL indicators, the 50% threshold was adopted as the tolerance.

For the MIN, MAX and AVG indicator family, preference functions were established assuming the value of 1 for the reference value and the value of 0 for the value outside the symmetrical tolerance range. In the case of the MIN, MAX, and AVG indicators, the reference (desired) value is 0 (zero deviation). Preference functions for an established tolerance (t_min_, t_max_) take the form (7):

(7) $$\mathrm{PREF}\left(x\right)=\{\begin{array}{cc} 0, & \;x<t_{\min}\;\mathrm{lub}\;x>t_{\max}\\ \;\frac{x+t_{\min}}{t_{\min}}, & \;x\in \left[t_{\min},\;0\right)\\ \;\frac{t_{\max}-x}{t_{\max}}, & \;x\in \left[0,t_{\max}\right] \end{array}$$

For the SIGMA and TOTALFAIL indicators, a one-sided preference function was applied, assuming the value 1 for the indicator value 0 and the value 0 for an indicator value above the tolerance limit. In the case of the SIGMA and TOTALFAIL indicators, the reference (desired) value is the value 0 (zero deviations and no measurements outside the tolerance limit). Thus, the preference functions for the established tolerance t take the form (8):

(8) $$\mathrm{PREF}\left(x\right)=\{\begin{array}{c} \;\frac{t\;-\;x}{t},\;x\in \left[0,\;t\right)\\ \;0,\;x\geq t \end{array}$$

The above-defined tolerance functions assume the value 0 outside the tolerance range. In the subsequent process of parameter optimisation, for the values obtained from the estimating models, a situation may occur in which (1) the values of SIGMA and TOTALFAIL are negative, or (2) sets of parameters are non-zero and are pairwise uncoupled, which leads to the zeroing of the global preference function for each set parameters. For this reason, a variation of the preference function was introduced by minimising the preference functions, assuming the value of 0.01 outside the tolerance intervals and the value (0.01, 1) within the tolerance interval.

Functions defined in this way will allow one to obtain a non-zero value of the global preference function in the parameter space, while achieving a low value for parameters resulting in predictions beyond the tolerance range for indicators. The minimising preference functions for the indicators MIN, MAX, AVG (Equation (9)) and SIGMA, TOTALFAIL (Equation (10)), take the form:

(9) $$\mathrm{PREF}\left(x\right)=\{\begin{array}{cc} 0.01, & \;x<t_{\min}\;\mathrm{or}\;x>t_{\max}\\ \;\frac{0.99x+t_{\min}}{t_{\min}}, & \;x\in \left[t_{\min},\;0\right)\\ \;\frac{t_{\max}-0.99x}{t_{\max}}, & \;x\in \left[0,t_{\max}\right] \end{array}$$

and

(10) $$\mathrm{PREF}\left(x\right)=\{\begin{array}{c} \;\frac{t\;-0.99x}{t},\;x\in \left[0,\;t\right]\\ \;0,\;x>t \end{array}$$

**Figure 5:**
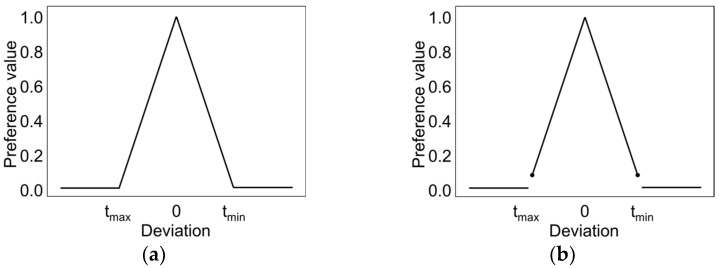

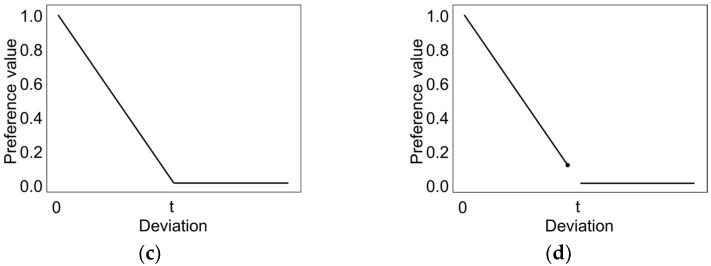
Graphs of (**a**) the preference function for the indicators MIN, MAX, AVG; (**b**) the minimising preference function for the indicators MIN, MAX, AVG; (**c**) the preference function for the SIGMA and TOTALFAIL indicators; (**d**) the graph of the minimising preference function for the SIGMA and TOTALFAIL indicators.

The preference function and minimising preference function for the indicators MIN, MAX, AVG, SIGMA and TOTALFAIL are presented graphically in Figure 5.

In the process of parameter optimisation, according to the procedure described above, a multivariate optimisation task was built for the quality preference function. This task was then solved using the grid search method. After determining the optimal parameters, the responses for individual indicators were calculated from regression models forecasting the preference function argument for a given indicator. After obtaining the optimal parameters, subsequent trials of the shear spinning process were used to validate the solution obtained and, if necessary, adjust the parameters. Validation was carried out in three technological trials. The parameter sets for each trial were established based on the optimisation results. After the forming tests, the products were subjected to visual quality inspection as well as dimensional and geometry inspection. After industrial validation of the solutions, a measure was introduced on the basis of which the results of the validation tests were compared with the assumptions. To compare the results of the P_i_ sample, the following measure of the mean relative change in absolute deviation was introduced to the established reference sample P_r_ for a certain subset of Q indicators (Equation (11)):

(11) $$d_{Q}\left(P_{i},P_{r}\right)\;≔\frac{1}{\left|Q\right|}\underset{q\;\in \;Q}{\sum }\frac{\left|q\left(P_{r}\right)\right|-\left|q\left(P_{i}\right)\right|}{\left|q\left(P_{r}\right)\right|}$$

where q(P_i_) is the value of the indicator q obtained in the trial P_i_. A function defined in this way makes it possible for the average and relative improvement of the indicator values for the sample P_i_, to be determined. The degree of improvement is considered to be the increase in the value of the indicators towards zero.

Calculations and analyses were performed with the use of the statistical packages available for Python and R languages (statsmodels and rpy2), as well as with the GOM Inspect software.

After the technological trials, the microstructure of the samples was examined. Samples were taken from zones **Z1**, **Z3** and **Z5** (Figure 4).

## 3. Results and Discussion

**Figure 6:**
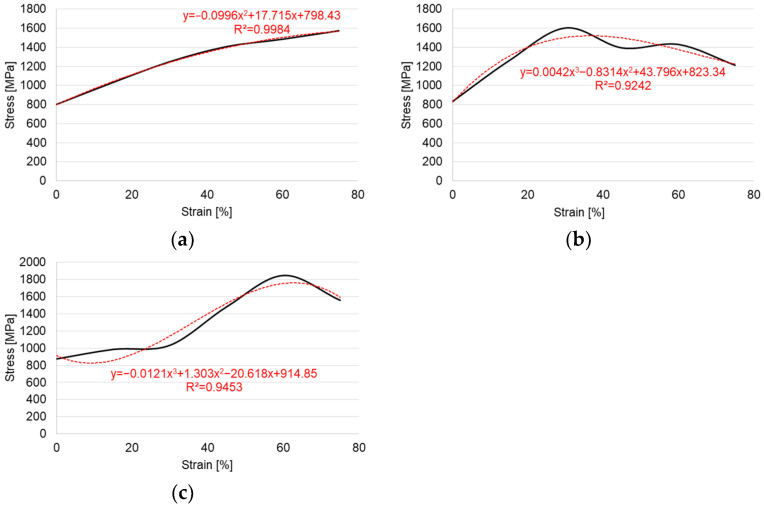
Stress–strain curves (**a**) for 0°, (**b**) 45°, and (**c**) 90°.

Figure 6 shows the results of research on the mechanical properties of the test material.

Table 5 presents the results of measurements of the test material anisotropy.

The results of the Lankford anisotropy coefficient calculations show that the sheet exhibit a high anisotropy. The R-value is almost twice as large for the sheet in the direction of 45° as for the sheet in the direction of 0°. The mean coefficient of normal anisotropy is $\bar{r}=1.075$. It should therefore be stated that the sheet has a low ability to be formed by stamping.

**Figure 7:**
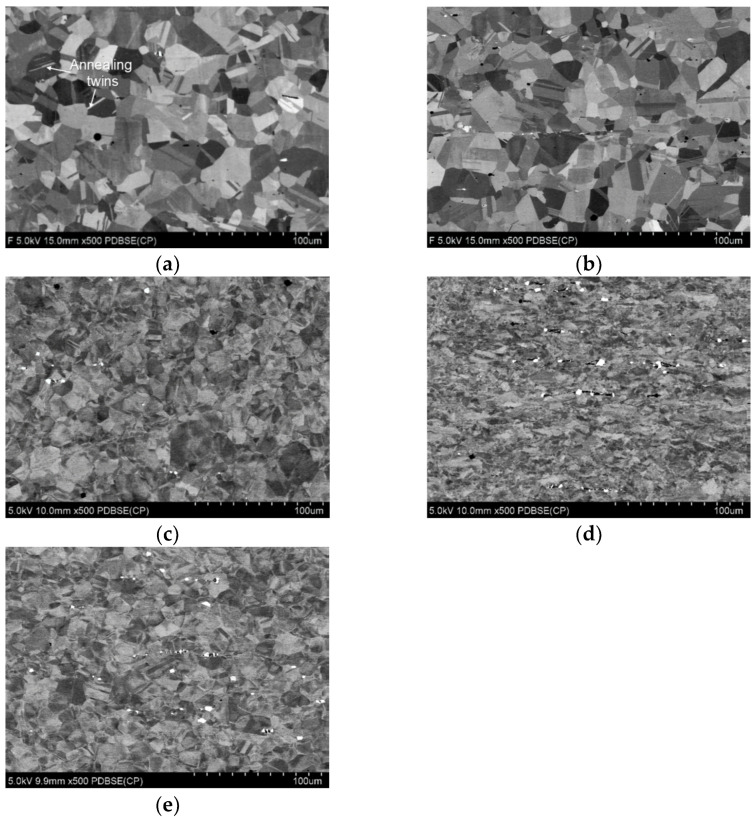
SEM images of the test material microstructure (**a**) cross section, (**b**) longitudinal section. SEM images of the microstructure of the material after deformation taken (**c**) from zone **Z1**, (**d**) zone **Z3**, and (**e**) zone **Z5**.

Figure 7 shows the results of the batch material microstructure (Figure 7a–b) analysis and after deformation (Figure 7c–e).

The mean area of the grains is minimally different in the case of samples taken in the parallel and perpendicular directions. The mean grain size of the samples taken in the perpendicular direction is 1.15% smaller than the mean grain size of the samples taken in the parallel direction. Conversely, the approximate grain diameter in the case of samples taken in the perpendicular direction is 1% larger than in the case of samples taken in the parallel direction. Annealing twins can be distinguished in the structure (Figure 7a). It is a characteristic element of the austenitic alloy structure. They are characterized by a lattice with low stacking-fault energy, which results in a high frequency of stacking errors, and thus resistance to plastic deformation.

The structure of the material after deformation is a structure characteristic of the material after intense deformation. The grains are elongated, the grain boundaries are not clear. The photos show strain localization which plastic deformation occurred. The average grain size for **Z5** (40% deformation) is lower by 46.59% compared to the average grain size of the batch material, for **Z3** (60% deformation) by 73.94%, while for **Z1** (deformation 10%) by 39.47%. The approximate grain diameter for **Z5** (40% deformation) is lower by 24% compared to the average grain diameter of the batch material, for **Z3** (60% deformation) by 44%, and for Z1 (10% deformation) by 19.5%.

Based on a visual assessment of the components, the forming trials were considered successful. All the products were sent for further research. The results of an overall comparison of the components surface and thicknesses with the CAD model and measurement data was generated and analysed according to the experiment plan.

**Figure 8:**
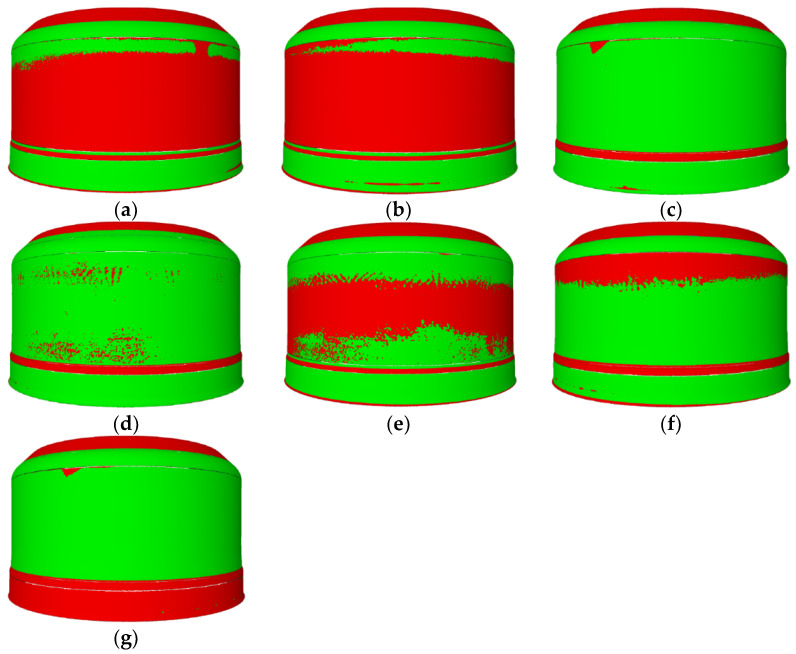
Areas of components after technological tests within given tolerances: P_0_ (**a**), P_1_ (**b**), P_2_ (**c**), P_3_ (**d**), P_4_ (**e**), P_5_ (**f**), and P_6_ (**g**).

Figure 8 presents maps of the zonal comparison of the thickness of the areas of products that meet the tolerances (green) and those not within the acceptable tolerance (red) together with the measurement data. Compatibility of the sheet thickness of the components within the tolerance limits in five zones (Z1–Z5) and compliance of the surfaces with the tolerance in two zones (Z1, Z6) were analysed. The measurement data for the zonal comparison of the sheet thickness in areas within the tolerance generated from the trials were used to build the models discussed later in this article.

In the next step, models were built which explain the influence of factors on individual quality criteria. Then an optimisation scheme was launched to return parameters that maximise the global preference function. An attempt was made to determine the influence of S, R and H factors on the quality of the final component (measured using indicators) and their interactions. The potential significant main effects and implicit interactions were determined according to the methodology of analysis of the results of the experimental plan $2_{\mathrm{III}}^{3-1}$. In the case of the plan considered, these pairs were (S, R:H), (R, S:H) and (H, S:R). Student’s T test was used to determine the significance of differences between the marginal means for the levels of the factors examined.

The dominant interaction was the interaction between the feed rate and the rotational speed of the spinning block S:R, defined in later analysis as the feed factor F := S/R. The main effects and interactions identified were used to determine regression models approximating the values of the indicators. S, R and H as well as their mutual interactions were significant factors influencing the values of the indicators. Linear regression models were fitted using the least squares method. The categorical factor H was included in the models using the basic dummy coding, and the coefficient of determination was adopted as a measure of fit. The explanatory variables were standardised.

Initial attempts to obtain correct models with linear S, R, H and F factors and their interactions did not bring the desired results; thus, it was decided to include non-linear factors and apply the following transformations: $f\left(x\right)=x^{-1},\;f\left(x\right)=x^{-2},\;f\left(x\right)=x^{-3},\;f\left(x\right)=x^{2},\;f\left(x\right)=x^{3},$ f(x)=arctan(x), f(x)=exp(x), and f(x)=log(x). Using such an extended class of factors, satisfactory models were adjusted for most indicators (coefficient of determination 0.7–1.0). Due to the relatively small size of the sample and the number of transformations of factors, a significant number of models that meet the criteria were obtained for many indicators. The selection of the final models was also guided by the evaluation of the trend and the complexity of the model. Models containing strong interactions with H were avoided due to the small number of trials with the value of H = 1. Moreover, models characterised by rapid growth of predicted value outside the approximation range were also avoided.

The regression models obtained, which estimated the indicator values, were used in the multivariate optimisation process which operates by maximising the global preference function. For each of the indicators, after considering the appropriate tolerances, the preference functions were determined in accordance with the relationships (7) and (8). Initial analysis of the models obtained revealed the need to correct the preference function such that the global preference function assumed non-zero values on a non-empty set of parameters. In addition, it was decided to investigate various optimisation variants-apart from the unconditional variant (UV), by zeroing the global preference function for values lying outside the tolerances, the conditional variant (CV) and the minimisation variants: unconditional (UMV) and conditional (CMV), were also analysed.

The conditional variants assume the assignment of a small preference value to the values of indicators outside the tolerance limits, in accordance with Equations (9) and (10). In this way, the possibility of the appearance of indicators with values outside the tolerance limits, when the values of most indicators are close to the preferred value, was allowed for. These variants are particularly important for indicators assuming a value outside the tolerance limits over all the process parameters. In such a situation, the preference function would assume a constant value, ignoring the variability of these indicators and making it impossible to compare their values for different sets of parameters. The minimisation variants solve this problem, allowing one to optimise the values of indicators beyond the given tolerances. In order to obtain minimisation variants, the set tolerances were modified by introducing the so-called minimisation tolerances. Their values were established at a level not lower than the maximum for positive tolerances, and at a level not lower than the minimum for negative tolerances, and the highest/lowest value obtained in the experimental trials $P_{1}-P_{5}$. In the optimisation carried out for such defined tolerances, the indicator values obtained were within the given tolerances or were not worse than those obtained in the experimental tests.

For the Q indicator, the set tolerance $t\;≔\left[t_{\min},t_{\max}\right]\;$ and the set R of the experimental results, the tolerances of the minimisation variant were defined as follows (Equation (12)):

(12) $$t^{\min}\;≔\left[\min\left(t_{\min},\min\left(R\right)\right),\max\left(t_{\max},\max\left(R\right)\right)\right]$$

The characteristics of the optimisation variants are presented in Table 6.

In addition, considering the regression approximation error, the tolerances were corrected for the deviation of the test results from the predicted values. This deviation was calculated as the root mean square of the residuals.

Basic tolerances were adopted for the area indicators in accordance with the technical specification of the component (Figure 4). For overall indicators, TH_G and SURF_G, a positive (negative) tolerance was determined as the maximum (minimum) of area tolerances. For the TOTALFAIL indicators, the 50% threshold was adopted as the tolerance.

The global preference function—the weighted geometric mean of the value of the preference function—set with relationship (3), takes as a parameter the validity of individual indicators. A varied range of validity allow for emphasis to be given to the optimisation of significant quality indicators, and in the case of similar preference values, parameters are selected for which higher preferences are achieved for indicators with a lower validity. In other words, changes in indicators with a higher validity have a greater impact on the global preference function. Four sets of validity were adopted, favouring the most important families of indicators-quality indicators for thickness measurements, quality indicators for surface measurements and quality indicators for the most validity zones Z1, Z3 and Z6. The validity in individual sets were set at the level of 10 for the preferred indicators, and at the level of 1 for other indicators. Additionally, a set of validity assuming equal significance of all indicators was considered. Defined sets of validity are presented in Table 7.

The Cartesian product S ×R ×H was adopted for the following ranges of process variables S∈[19, 46], R∈[215, 335], H∈{0,1} as the discrete design space of the process, constituting the search space for the grid search scheme. The unit resolution of the ranges was also determined (a step with a value of 1). The adopted ranges are close to the ranges determined by the parameters for which the experiments were performed. The lack of these assumptions could result in an erroneous location of the optimal parameters because the regression models obtained may contain large approximation errors in the values of the factors for which the fit was obtained. For this reason, a condition was also imposed on the value of F ≔ S/R requiring that F∈[0.07, 0.18].

Due to the acceptable time complexity of the method for the adopted ranges of parameters, an exhaustive search approach was chosen. For each vector of the design space, the value of the global preference function was calculated, and a set of process parameters was determined that maximised the overall value of preferences. In total, sixteen optimisation sub-processes were carried out (for each optimisation variant and set of validity), allowing the optimal parameters for variously defined priorities and quality requirements to be obtained.

**Figure 9:**
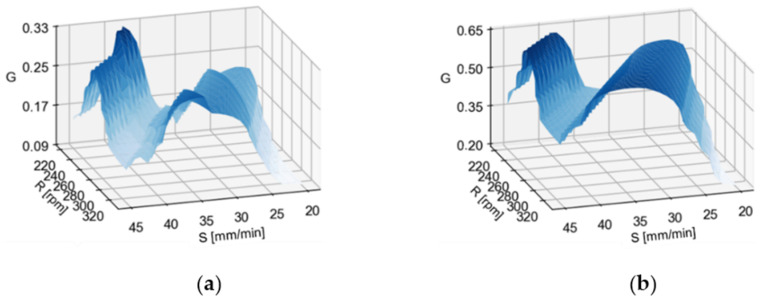
Graphs of the global preference function for (**a**) the variant CV and weights W_ALL and (**b**) CMV and weights W_Z; with H = 1 fixed.

Figure 9 shows the global preference function for selected optimisation variants and sets of validity. In all cases, the local minima and maxima are located in similar areas; the functions tend to adopt maxima for values close to the boundary values of the F-parameter as a result of the strong dependence between regression models and the F-parameter. Different characteristics of the function can be noted. Regular variants have discontinuous preference functions and are reflected in an irregular graph, high variability of functions and jumps in values, while minimisation variants produce in a more regular and smoother course.

**Figure 10:**
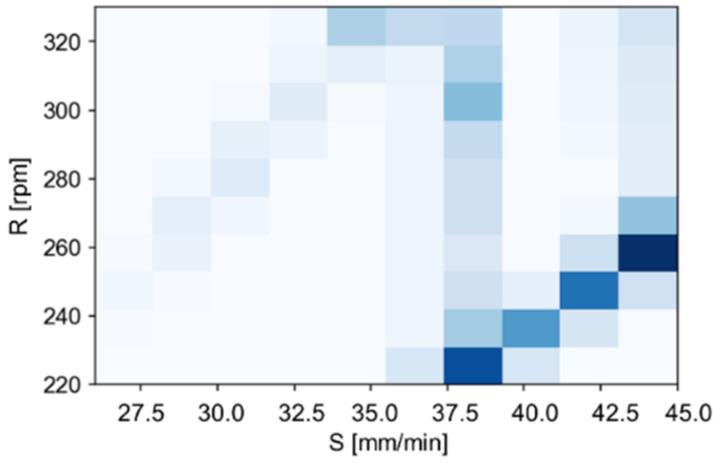
Histogram of the most common sets of parameters in the 5% of the results that were the best (the darker the colour, the higher the frequency of occurrences).

As a result of the high resolution of the design space, the sets of parameters for which the highest values of the global preference function were achieved, were generally spread within a few units for each parameter. It was necessary to aggregate the results to determine the dominant ranges of parameters and, as a result, optimal sets of parameters for each of the tested variants. For this purpose, an examination was made of the frequency of occurrence of particular sets and ranges of parameters in the 5% of the results that were the best (values of the global preference function). In order to illustrate and evaluate the correctness, histograms of the number of parameter sets were prepared (Figure 10).

**Figure 11:**
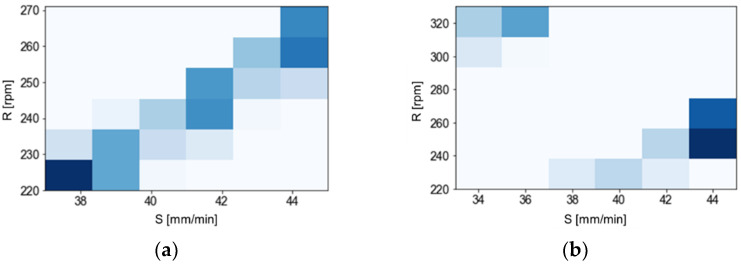
Histogram of (**a**) the most common sets of parameters for the best 5% of the CMV results and the W_ALL weight set and of (**b**) the best 5% of the CMV results and the TH weight set (the darker the colour, the higher the frequency of occurrences).

Figure 11 presents the aggregated frequency for all sixteen optimisation variants, revealing the dominant influence of parameters from the ranges: S∈[38, 45] and R∈[240, 270]. The H parameter was set at level 1, as occurring in most of the tested parameter sets, or having complementary sets (differing only in the H-value) in the 5% of the results that were the best that were analysed.

Table 8 presents the dominant sets of parameters for each optimisation process carried out for various optimisation variants and sets of weights. For the conditional variant, it was not possible to obtain parameters resulting in the values of all indicators within the required tolerances.

Finally, three sets of parameters were selected for which the highest preference values, i.e., the best predicted product quality: $V_{1}=\left[45,\;259,\;1\right]$, $V_{2}=\left[39,\;225,\;1\right]$ and $V_{3}=\left[45,\;325,\;1\right]$, were obtained. The selected parameters predict the improvement of the results measured in the number of indicators whose values are within the given tolerances, and the improvement of the values of individual indicators. The predicted compliance with the tolerance for the quality indicators was evaluated. A comparison with the experiments performed predicts an increase in the number of compatible indicators by 11–53%. The optimal sets of process parameters obtained: $V_{1}=\left[45,\;259,\;1\right]$, $V_{2}=\left[39,\;225,\;1\right]$, and $V_{3}=\left[45,\;325,\;1\right]$, were tested in verifying technological trials. Trial V_3_ failed. It was impossible to uninstall a component from the spinning block. Components produced by trials V_1_ and V_2_ were sent for further research. After 3D scanning the results of an overall comparison of the components surface and thicknesses with the CAD model and measurement data was generated and analysed according to the experiment plan.

**Figure 12:**
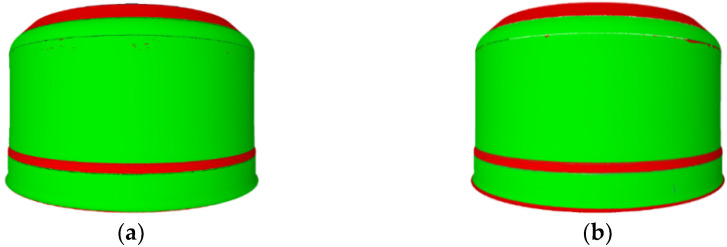
Areas of components after technological tests within given tolerances: $V_{1}$ (**a**); $V_{2}$ (**b**).

Figure 12 presents maps of the zonal comparison of the thickness of the areas of products that meet the tolerances (green) and those not within the acceptable tolerance (red) together with the measurement data. Compatibility of the sheet thickness of the components within the tolerance limits in five zones (**Z1**–**Z5**) and compliance of the surfaces with the tolerance in two zones (**Z1**, **Z6**) were analysed.

Values of indicators obtained in trials V_1_ and V_2_ were compared with regression models obtained in the optimisation process. The best compliance for the individual indicators was achieved for the second and third zones, which may result from the characteristics of those areas-they have simple geometry. For some models, the results obtained suggest the required correction of the model had occurred with the need to recognise certain experimental results as outliers.

In order to test the correctness of the approach applied, the results obtained after the validation tests were compared with the theoretical predictions and assumptions of the optimisation process. A comparison was made between the theoretical and resulting values of individual indicators and sets of indicators within the required tolerance ranges. The mean, relative change in the absolute value of the deviation was adopted as a measure of the estimation error (Equation (11)). Good compliance was obtained between the values obtained at the optimisation stage and the actual test results.

**Figure 13:**
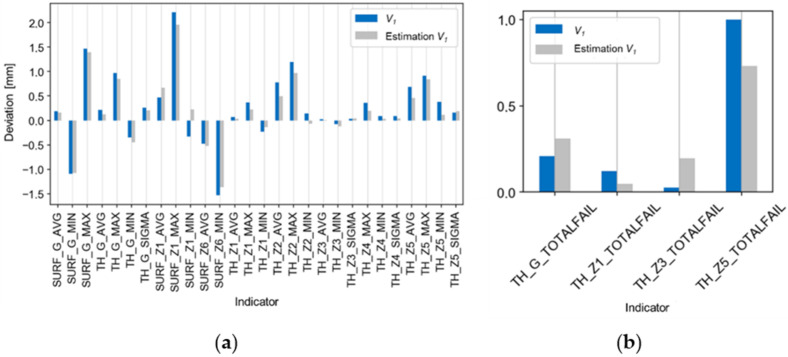
Comparison of the estimate of the results of the V_1_ trial (based on regression models) with the actual results of the trial for the indicators of (**a**) deviations and (**b**) tolerances.

**Figure 14:**
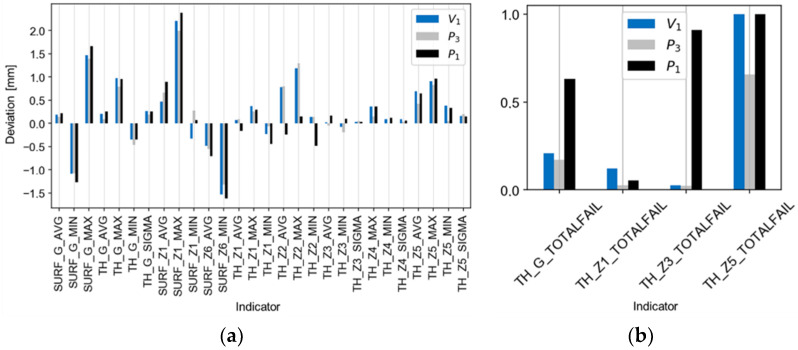
Comparison of the results of the V_1_ trial with the best and worst results of the experiments P_3_ and P_1_ for the indicators of (**a**) deviation and of (**b**) tolerance.

**Figure 15:**
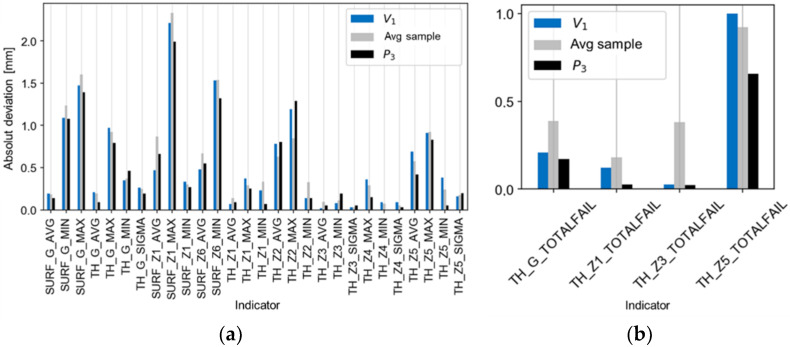
Comparison of the absolute deviations of the results of the V1 trial with the absolute best and average results of the experiment for the indicators of (**a**) deviation and of (**b**) tolerance.

Figure 13, Figure 14 and Figure 15 present comparisons of the results for the V_1_ trial with the estimates, (Figure 13a,b), with the best and worst results of the technological tests (Figure 14a,b), and with the absolute deviations of the results for the V_1_ trial (Figure 15a,b). There is a good agreement found in the values and directions of the deviations (Figure 13a,b). The results of the validation tests were compared with the results of the P_3_ test, for which the best results were found (eighteen indicators compliant with the tolerance limits). The mean, relative improvement of the absolute deviation (Figure 15a,b) for indicators outside the tolerance range compared to the P_3_ trial was −10% for the V_1_ trial.

**Figure 16:**
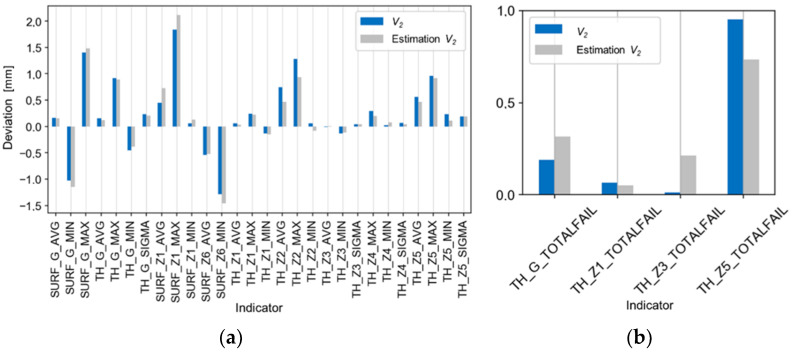
Comparison of the estimates of the results of the V2 trial (based on regression models) with the actual test results for the indicators of (**a**) deviations and of (**b**) tolerance.

**Figure 17:**
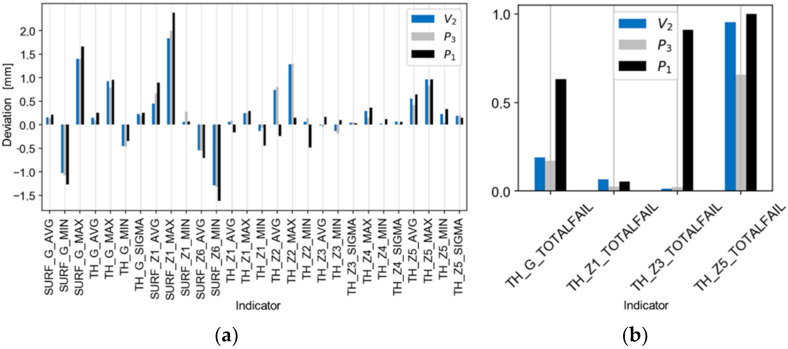
Comparison of the results of the V2 trial with the best and worst results of the experimental trials P3 and P1 for the indicators of (**a**) deviation and of (**b**) tolerance.

**Figure 18:**
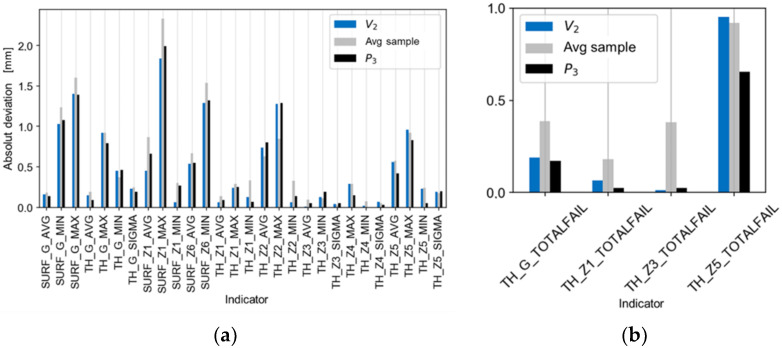
Comparison of the absolute deviations of the results of the V2 trial with the absolute best and average results of the experimental trials for the indicators of (**a**) deviation and of (**b**) tolerance.

Figure 16, Figure 17 and Figure 18 show the comparisons of the results of the V_2_ trial with the estimates (Figure 16a,b), with the five best and worst results of the technological tests (Figure 17a,b), and with the absolute deviations of the results of the V_2_ trial (Figure 18a,b). There is good agreement in the values and directions of the deviations (Figure 16a,b). The average, relative improvement of the absolute deviation (Figure 18a,b) for indicators outside the tolerance range compared to the P_3_ trial was 6% for the V_2_ trial.

The compliance of the actual values of the indicators was compared with the forecasts obtained from regression models. The values obtained in the trials V_1_ and V_2_ for the majority of indicators are correctly predicted by the models, keeping the direction and trend of the deviations (Figure 11a,b and Figure 16a,b). For the predicted number of indicators compliant with the tolerance limits, the prediction accuracy for trials V_1_ and V_2_ was 94% and 97%, respectively. For trials V_1_ and V_2_, the number of indicators with values within the set tolerance increased by 11% (to twenty indicators within the desired range) for trial V_2_ and decreased by 7% for trial V_1_ (seventeen indicators within the tolerance range).

## 4. Conclusions

Authors should discuss the results and how they can be interpreted from the perspective of previous studies and of the working hypotheses. The findings and their implications should be discussed in the broadest context possible. Future research directions may also be highlighted.

Optimisation schemes for the shear spinning process of Inconel 625 nickel superalloy were analysed in search of a method permitting for effective minimisation of the global response function. The most important criteria in selecting an optimisation scheme are the complexity of the problem, the topology of the process design space and the class of the cost function. Due to the acceptable size of the problem and the discrete parameter space, the grid search method was adopted.

On the basis of multidimensional optimisation, sets of optimal parameters of the shear spinning process with laser heating of Inconel 625 nickel superalloys were selected. For the predicted number of indicators compliant with the tolerance, the prediction accuracies for parameter sets V_1_ and V_2_ were 94% and 97%, respectively. The values obtained in the trials V_1_ and V_2_ for most indicators are correctly predicted by the models, keeping the direction and trend of the deviations. Inaccuracies in the results could be the result of the approximation methods used, the quality of model fit, and the measurements carried out by the operator.

The methods used proved to be effective in the design, evaluation and verification of the shear spinning process. They enable excellent control of process variables and the development of the degree of accuracy in predicting the effects of the forming process, along with the number of trials performed. The advantage of the method of process optimisation adopted is the property of self-regulation. As the number of verification tests performed increases and they are included in the input data set, the accuracy of model prediction increases. This results in more precise estimates in future trials until they converge and reach the optimum. The method allows for optimisation without direct access to tools and details of the process, which may be confidential information, unlike FEM, where the user must have a full description, including specifications and models. In addition, the advantage of the adopted method is the lower cost of the creation of such solution, than in the case of FEM. FEM requires appropriate substantive advancement of the personnel, while its implementation usually involves expensive, commercial software. Conversely, the adopted optimisation method used free libraries, and the level of advancement is not as demanding as in the case of FEM.

## Figures and Tables

**Table 1:** Results of the experiment $T_{1}$ and $T_{2}$.

T_1_				T_2_			
	A1	A2	Marginal mean		A1	A2	Marginal mean
B1	10	10	10	B1	10	20	15
B2	20	20	20	B2	20	10	15
Marginal mean	15	15		Marginal mean	15	15	

**Table 2:** Two-level orthogonal design of the experiment for three factors.

No	X1	X2	X3
1	1	1	1
2	1	−1	−1
3	−1	1	1
4	−1	−1	−1

**Table 3:** Levels of factors.

Factor	Level 1	Level 2
S	$S_{1}=1.2S_{0}=36\;\left(\mathrm{mm}/\min\right)$	$S_{2}=1.5S_{0}=45\;\left(\mathrm{mm}/\min\right)$
R	$R_{1}=1.15R_{0}=253\;\left(\mathrm{rpm}\right)$	$R_{1}=1.5R_{0}=330\;\left(\mathrm{rpm}\right)$
H	1	0

**Table 4:** The trail plan.

Trial	S (mm/min)	R (rpm)	H	f (S/R) (mm/obr)
$P_{0}$	30	220	0	0.136
$P_{1}$	36	253	0	0.142
$P_{2}$	36	330	1	0.109
$P_{3}$	45	253	1	0.177
$P_{4}$	45	330	0	0.136
$P_{5}$	24	253	0	0.095
$P_{6}$	20	253	0	0.080

**Table 5:** Lankford coefficient for the test material.

Direction	Lankford Coefficient
0°	0.68
45°	1.25
90°	1.12

**Table 6:** Optimization variants.

	Out-of-Tolerance Value of Preference: 0	Preference Value out of Tolerance: 0.01
Regular tolerances	UV	CV
Minimisation tolerances	UMV	CMV

**Table 7:** The weight sets used to optimise shear forming parameters.

Set of Weights	Preferred Indicators (Validity 10)	Other Indicators (Validity 1)
W_ALL (all indicators)	TH_G, TH_Z, SURF_G, SURF_Z	
W_TH (thickness indicators)	TH_G, TH_Z	SURF_G, SURF_Z
W_SURF (surface indicators)	SURF_G, SURF_Z	TH_G, TH_Z
W_Z (indicators of zones **Z1, Z3, Z6**)	TH_Z1, TH_Z3, TH_Z6, SURF_Z1, SURF_Z6	TH_G, TH_Z2, TH_Z4, TH_Z5, SURF_G

**Table 8:** Dominant sets of parameters in the optimisation processes performed.

	W_ALL	W_TH	W_SURF	W_Z
UV				
CV	[1, 39, 225]	[1, 39, 225]	[39, 225, 1]	[39, 225, 1]
UMV	[45, 266, 1]	[45, 266, 1]	[45, 266, 1]	[45, 266, 1]
CMV	[37, 330, 1]	[37, 330, 1]	[37, 330, 1]	[37, 330, 1]

## Data Availability

The data presented in this study are available on request from the corresponding author.
